# Wax deposition is vital for thermotolerance in rice

**DOI:** 10.1016/j.xplc.2025.101317

**Published:** 2025-03-15

**Authors:** Kamankshi Sonkar, Amarjeet Singh

**Affiliations:** National Institute of Plant Genome Research, New Delhi 110067, India

## Main text

Rice is a staple food and a vital source of nutrition for over half of the world’s population. Unfortunately, it is highly susceptible to heat stress during both vegetative and reproductive developmental stages. As predicted, in current climate change and global warming patterns, even a 1°C rise in environmental temperature could decrease cereal production by 6%–7% (Lesk et al., 2016). This poses a serious threat to the food security of the ever-growing global population. To prepare for this environmental emergency, there is a dire need for enhancing the thermotolerance of this important crop plant.

Different mechanisms have been demonstrated for thermotolerance in plants, including cell wall remodeling, Ca^2+^ signaling, transcriptional regulation of heat shock proteins and heat shock factors, and protein modeling (Kan et al., 2023). In addition, molecular mechanisms such as transcript processing, protein translation, post-translational regulation, and epigenetic modifications are proposed to underlie thermotolerance in rice (Li et al., 2023). However, the molecular mechanisms of thermotolerance in rice are poorly understood. The deposition of cuticular wax is a protective adaptation in plants, as it prevents excessive water loss through transpiration under water-limiting conditions, such as drought and heat (Lewandowska et al., 2020). Heat stress has been shown to simultaneously hamper fatty acid elongation and wax biosynthesis and promote cuticular permeability in plants, leading to water loss. Deposition of a cuticular wax layer lowers plant canopy temperature and minimizes water loss (Kan et al., 2022). Moreover, a wax layer acts as a protective barrier against invading pathogens and insect herbivores. These attributes of wax deposition may improve the thermotolerance of plants.

To date, a few key quantitative trait loci (QTLs) have been identified for thermotolerance in rice. Thermo-tolerance 1 (TT1), first identified in the African rice cultivar *Oryza glaberrima*, codes for an α2 subunit of the 26S proteasome that targets ubiquitinated denatured proteins for degradation. Both the TT1-harboring nearly isogenic line and TT1-overexpressing lines of cultivated rice display enhanced thermotolerance (Li et al., 2015). Another QTL, ALKALI-THERMAL TOLERANCE 1/2 (ATT1/2), which encodes GA20 oxidases, has been shown to improve alkali–thermal tolerance by fine-tuning the endogenous level of gibberellin in rice (Guo et al., 2025). Recently, two additional QTLs, TT2 (Kan et al., 2022) and TT3 (Zhang et al., 2022), have been identified in rice. TT3 consists of two genes: *TT3.1*, which encodes a plasma membrane-localized E3 ligase, and *TT3.2*, which encodes a chloroplast precursor protein. Upon perception of heat stress, TT3.1 (proposed as a thermosensor) translocates from the plasma membrane to endosomes, where it ubiquitinates TT3.2, leading to its vacuolar degradation. A reduced level of TT3.2 protein is desirable for protecting thylakoids from heat stress (Zhang et al., 2022). Thus, the interaction between TT3.1 and TT3.2 is crucial for improved thermotolerance.

**Figure 1:**
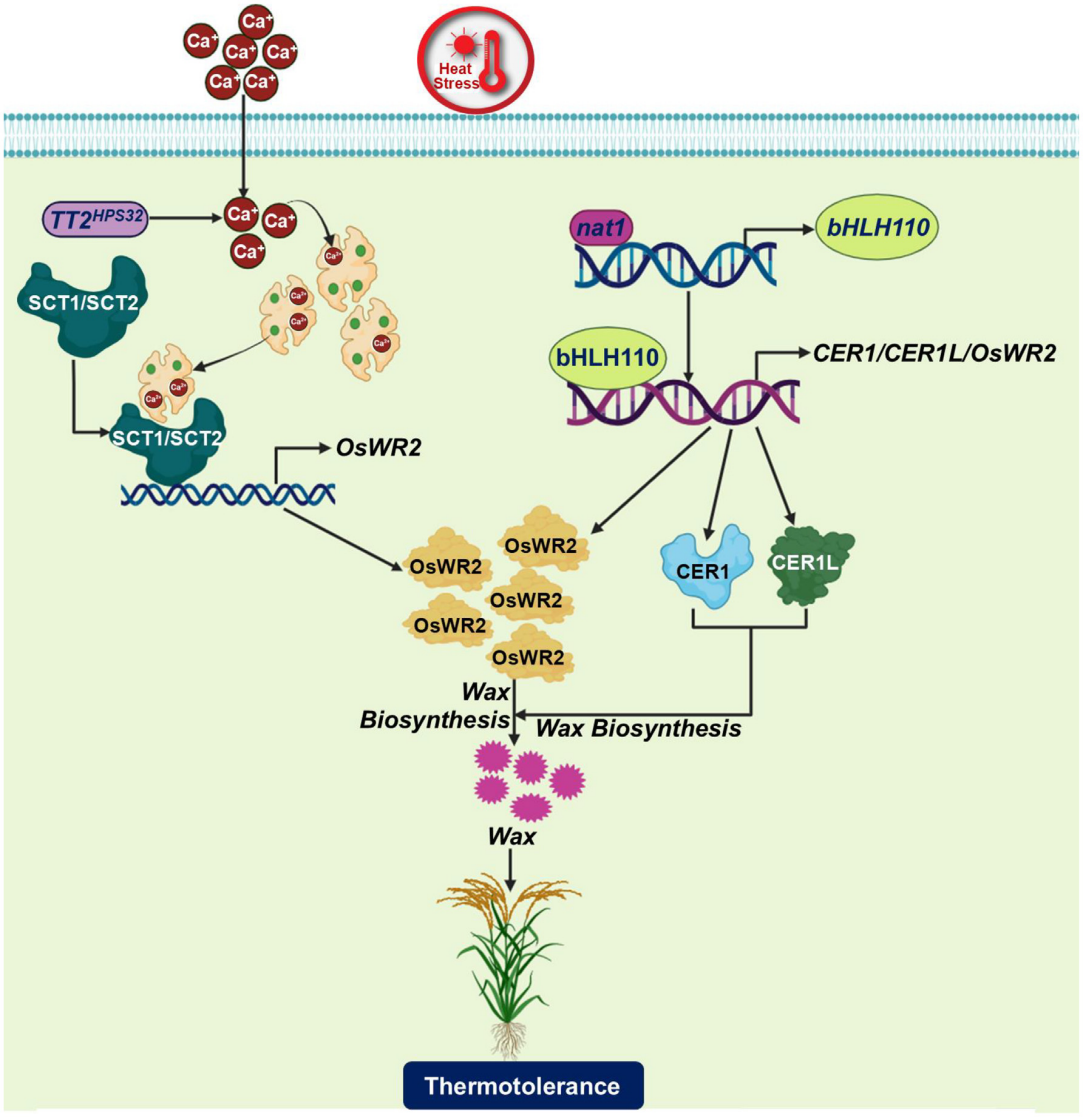
A model depicting the molecular mechanism of wax deposition and thermotolerance in rice**.** Heat stress- induced elevation in cytosolic Ca^2+^ level is reduced by the natural TT2 allele, *TT2*^*HPS32*^, as reported by Kan et al. (2022). When cytosolic Ca^2+^ levels are low, CaM remains insufficiently activated, hampering its ability to bind with SCT1/SCT2. This interruption prevents the suppression of SCT1/SCT2 activity by CaM, which typically occurs in the presence of the normal TT2 allele. As a result, SCT1/SCT2 is activated, driving optimal expression of *OsWR2* and promoting robust wax biosynthesis. Lu et al. (2025) demonstrated that in the *nat1* mutant, which lacks the negative regulator of thermotolerance *NAT1*, the inhibition of bHLH110 expression is removed. Once activated, bHLH110 enhances the expression of wax biosynthesis-related genes such as *CER1*, *CER1L*, and *OsWR2*, thereby significantly enhancing wax biosynthesis. Collectively, both pathways contribute to substantial wax deposition, enhancing thermotolerance in rice.

TT2 (GS3) encodes a G protein γ (Gγ) subunit. Sequence analysis of TT2 suggests that its natural allele *TT2*^*HPS32*^ harbors a SNP (C165A) that causes a premature stop codon in its open reading frame (Kan et al., 2022). Thus, the natural allele *TT2*^*HPS32*^ functions as a loss-of-function mutant. Plants expressing *TT2*^*HPS32*^ display enhanced wax deposition under heat stress and increased thermotolerance, indicating that TT2 is a negative regulator of wax biosynthesis and thermotolerance in rice. A normal *TT2* gene (with no premature stop codon) is essential for heat-triggered elevation of cytosolic Ca^2+^ levels. This increase in cytosolic Ca^2+^ is decrypted by a calmodulin (CaM)-sensing Ca^2+^ transcription factor (SCT) module. High Ca^2+^ levels promote the binding of CaM to SCT1/SCT2, thereby suppressing SCT1/SCT2 activity and in turn, reducing the expression of its target genes, particularly *wax synthesis regulatory 2* (*OsWR2*). Consequently, there is reduced wax retention and decreased thermotolerance under heat stress (Kan et al., 2022). Rice plants carrying the natural allele TT2^HPS32^ show an impaired heat-triggered Ca^2+^ level increase, hampering the binding of CaM to SCT1/SCT2 and thus maintaining normal SCT1/SCT2-regulated expression of OsWR2 and sufficient wax content (Figure 1).

Recently, Lu et al. (2025) discovered a novel NAT1–bHLH110–CER1/CER1L module that also regulates wax biosynthesis for enhanced thermotolerance in rice. Under heat stress, the bHLH110 transcription factor acts as a positive regulator of two homologous fatty aldehyde decarbonylase genes, *ECERIFERUM1* (*CER1*) and *CER1-like* (*CER1L*). These genes are involved in the biosynthesis of very-long-chain alkanes, which are important for wax formation in rice (Islam et al., 2009). Negative regulator of thermotolerance 1 (NAT1), a C_2_H_2_ transcription factor, has been found to directly inhibit bHLH110 expression. Thus, NAT1 inhibits wax biosynthesis and reduces thermotolerance in rice by suppressing bHLH110–CER1/CER1L activity (Lu et al., 2025). Conversely, in the edited mutant *nat1*, the suppression of bHLH110 expression is removed. Activated bHLH110 increases *CER1/CER1L* expression, thereby promoting wax biosynthesis and thermotolerance (Figure 1). Notably, the NAT1–bHLH110–CER1/CER1L module enhances rice thermotolerance during both vegetative and reproductive growth. In addition, no alterations in *TT2–SCT1/SCT2* expression were observed in *nat1/bhlh110* mutants and NAT1/bHLH110-overexpressing plants, suggesting that the NAT1–bHLH110–CER1/CER1L and TT2–CaM–SCT1/SCT2 pathways are independent (Lu et al., 2025). However, OsWR2 appears to be a common downstream target in both pathways, as its expression was decreased in the *bhlh110* mutant and increased in bHLH110-overexpressing plants. Moreover, bHLH110 binds to the OsWR2 promoter and regulates its activity (Lu et al., 2025). This suggests that the NAT1–bHLH110–CER1/CER1L and TT2-CaM-SCT1/SCT2 pathways may work in tandem to regulate wax biosynthesis via OsWR2, particularly under heat stress.

Heightened stress tolerance often comes at the cost of yield penalties in crops. This is evident in previously engineered rice plants with improved thermotolerance (Mickelbart et al., 2015). Interestingly, enhanced thermotolerance conferred by the NAT1–bHLH110–CER1/CER1L and TT2–CaM–SCT1/SCT2 modules does not result in yield penalties. The natural allele TT2^HPS32^ exhibited no growth or yield penalties under normal conditions. In fact, TT2^HPS32^ exhibited improved 1000-grain weight compared to its isogenic control NIL-TT2^HJX^. Notably, grain production was 54.7% higher in TT2^HPS32^ plants than in NIL-TT2^HJX^ plants under heat stress (Kan et al., 2022). Similarly, three rice varieties with an edited *nat1* locus demonstrated improved thermotolerance without any growth or yield penalties under normal conditions. Importantly, *nat1* rice plants displayed an increased seed-setting rate and grain yield (Lu et al., 2025). This indicates that thermotolerance via manipulation of the wax biosynthesis pathway does not interfere with the developmental process in rice. This phenomenon could be explained by the expression pattern of *NAT1*, which was strongly induced in rice seedlings 30 min after exposure to heat. Tissue-specific expression analysis showed that *NAT1* was preferentially expressed in roots, stems, and developing endosperms under normal conditions. Notably, the basal expression level of NAT1 was low, which may explain the lack of growth or developmental defects and any noticeable yield penalties in the edited *nat1 mutant*. Thus, targeted gene editing of a negative regulator of abiotic stress could be a potential approach to enhance abiotic stress tolerance in rice and other crops without significant yield loss under normal growth conditions.

In summary, optimum cuticular wax biosynthesis has emerged as a vital adaptive mechanism under heat stress in rice. The NAT1–bHLH110–CER1/CER1L and TT2-CaM-SCT1/SCT2 modules are key in imparting enhanced thermotolerance without any yield penalties in rice. Several components of these modules, such as G protein γ subunits, SCT1/SCT2 (which belong to the CAMTA family), and NAT1, are highly conserved in other heat stress-prone cereal crops, including maize, sorghum, and wheat (Sun et al., 2018; Lu et al., 2025). Therefore, the investigation and engineering of these regulatory modules could also enhance thermotolerance in these crops. Thus, these candidate genes are of great biotechnological importance and will be valuable resources for breeding thermotolerant crop varieties through traditional methods or modern gene-editing technologies like CRISPR-Cas. Importantly, as layer of cuticular wax prevent the water loss, wax deposition will likely protect crop plants under other water-limiting environmental conditions such as drought, osmotic stress, and freezing. Moreover, as suggested by earlier reports, a wax layer may also protect plants from invading pathogens and herbivores. In the current scenario of climate change, plants face a multitude of abiotic stresses as well as combinations of biotic and abiotic stresses. Thus, optimal wax biosynthesis and deposition could be vital for generating stress tolerant and high-yielding “climate-smart” crops. It will be crucial to identify more downstream target genes of transcription factors like SCT1/SCT2, NAT1, and bHLH110. These targets could include other genes involved in wax biosynthesis besides OsWR2 and CER1/CER1L or key regulators of stress response and plant development. These unknown targets may contribute to thermotolerance and grain development in rice through diverse mechanisms, opening new avenues for future investigations and functional characterization of these targets to unearth novel thermotolerance mechanisms in crop plants. In the future, engineering crops using these potential genes, either individually or in combinations, may facilitate the development of highly thermotolerant cereal varieties without yield penalties, thereby helping to prevent crop yield losses due to drastic climate change and global warming.

## Funding

We are grateful for the financial support from the BRIC-NIPGR core research grant. A.S. acknowledges a research grant from the Anusandhan National Research Foundation (ANRF), 10.13039/100020373Government of India (grant CRG/2021/000694). K.S. thanks the Council of Scientific and Industrial Research (CSIR), India, for research fellowships. The authors acknowledge the DBT (Department of Biotechnology) eLibrary Consortium (DeLCON) for providing e-resources.

## Acknowledgments

No conflict of interest is declared.
